# Paradigm versus paradox on the prairie: testing competing stream fish movement frameworks using an imperiled Great Plains minnow

**DOI:** 10.1186/s40462-022-00306-9

**Published:** 2022-02-22

**Authors:** Zachary D. Steffensmeier, Maeghen Wedgeworth, Lauren Yancy, Noah Santee, Shannon K. Brewer, Joshuah S. Perkin

**Affiliations:** 1 Department of Ecology and Conservation Biology, Texas A&M University, College Station, TX USA; 2 Ecology and Evolutionary Biology Program, Texas A&M University, College Station, TX USA; 3 Oklahoma State University, Stillwater, OK USA; 4 U.S. Geological Survey, AL Cooperative Fish and Wildlife Research Unit, Auburn University, Auburn, AL USA

**Keywords:** Drift paradox, Pelagic-broadcast spawning, Restricted movement paradigm, Colonization cycle hypothesis, Dispersal, Conservation biology

## Abstract

**Background:**

Movement information can improve conservation of imperiled species, yet movement is not quantified for many organisms in need of conservation. Prairie chub (*Macrhybopsis australis*) is a regionally endemic freshwater fish with unquantified movement ecology and currently considered for listing under the Endangered Species Act. The purpose of this study was to test competing ecological theories for prairie chub movement, including the colonization cycle hypothesis (CCH) that posits adults must make upstream movements to compensate for downstream drift at early life stages, and the restricted movement paradigm (RMP) that describes populations as heterogeneous mixes of mostly stationary and few mobile fish.

**Methods:**

We tagged prairie chub with visible implant elastomer during the summer (May–August) of 2019 and 2020 to estimate net distance moved (m) and movement rate (m/d). We tested the hypotheses that observed prairie chub movement would be greater than expected under the RMP and that prairie chub movement would be biased in an upstream direction as predicted by the CCH.

**Results:**

We tagged 5771 prairie chub and recaptured 213 individuals across 2019 and 2020. The stationary and mobile components of the prairie chub population moved an order of magnitude further and faster than expected under the RMP during both years. However, we found only limited evidence of upstream bias in adult prairie chub movement as would be expected under the CCH.

**Conclusions:**

Our findings are partly inconsistent with the RMP and the CCH, and instead closely follow the drift paradox (DP), in which upstream populations persist despite presumed downstream drift during early life stages and in the apparent absence of upstream bias in recolonization. Previous mathematical solutions to the DP suggest organisms that experience drift maintain upstream populations through either minimization of drift periods such that small amounts of upstream movement are needed to counter the effects of advection or increasing dispersal regardless of directionality. We conclude that the resolution to the DP for prairie chub is an increase in total dispersal and our results provide insight into the spatial scales at which prairie chub conservation and management may need to operate to maintain broad-scale habitat connectivity.

**Supplementary Information:**

The online version contains supplementary material available at 10.1186/s40462-022-00306-9.

## Background

Understanding movement ecology of aquatic organisms has the potential to advance conservation and management of water resources [1]. Despite this potential, knowledge of movement by organisms was historically underrepresented in environmental management decisions for nongame species until recently when applied movement ecology emerged as a research framework [2–4]. For example, Fraser et al. [5] found that movement ecology information was incorporated into conservation planning most of the time when such data were available, but movement information was unknown for some at risk species. Allen and Singh [6] developed a five-step framework for integrating movement ecology into conservation planning, beginning with the measurement of movement attributes and extending to assessing organism effects on ecosystems, how this information can be incorporated into management, implementation of management actions, and finally evaluation of actions. Although this framework will provide beneficial information for animal conservation across taxa and ecosystems, it is particularly needed for imperiled stream fishes [2]. Specifically, there is a need to better understand the scales at which non-game or otherwise non-economically important stream fishes complete their life histories so that this information can be integrated into management and conservation actions [7].

Fish movement in streams received considerable attention during the past 70 years. Early works on stream fish dispersal documented largely restricted movements characterized by most individuals remaining near the site of tagging when recaptured [8, 9]. During this same early period, Funk [10] reported that fish populations in streams in Missouri, United States were composed of mixtures of sedentary fish that did not move far from the tagging location and mobile fish that moved greater distances. Despite observations by Funk [10], Gerking’s [9] conclusion of restricted movement by stream fishes was the prevailing regime for more than 30 years. Gowan et al. [11] reviewed fish movement literature and coined the term restricted movement paradigm (RMP) to emphasize the prevailing pattern of little movement by stream fishes. However, in the same work that derived the RMP, Gowan et al. [11] emphasized that stream fish movement studies focused too narrowly on habitats in which fish were marked and that fish populations were generally more mobile than reported. Rodriguez [12] later suggested that studies critical of the RMP were focused too heavily on a subset of Salmonidae fishes with high mobility; thus, the RMP was incomplete because it only acknowledged stationary fishes while ignoring mobile components of populations. Collectively, these works pointed to stream fish movement being heterogeneous and consisting of both “stationary” or “slow-moving” individuals and “mobile” or “fast-moving” individuals [13]. The signals of heterogeneous populations composed of stationary and mobile individuals (i.e., leptokurtic movement distributions) formed the basis of contemporary stream fish dispersal models [14]. In particular, Radinger and Wolter [14] used two overlapping normal distributions, one with a taller peak at zero movement (stationary fish) and a second with a shorter peak but broader base (mobile fish) to conduct a meta-analysis of movement by 40 species of stream fishes. Radinger and Wolter [14] developed predictions for stream fish movement under this new implementation of the RMP based on fish size, stream size, fish caudal fin aspect ratio (A = height^2^/surface area), and the length of time fish are at large (i.e., time between tag and recapture). These predictions are validated for several species including banded sculpin [15] and plains killifish [16]. However, tests of the RMP on a broader range of fishes are ultimately needed to determine if this theoretical framework is broadly applicable or subject to context dependencies.

A concept contrary to the RMP is the “colonization cycle hypothesis” (CCH), which posits that stream organisms that experience some degree of drift must move upstream to compensate for downstream displacement [17, 18]. The CCH predicts that most of the adult population must move a net distance upstream based on the downstream displacement of ova and larvae during drift. However, the CCH does not explain the upstream persistence of non-aerial adult macroinvertebrates that apparently do not make mass upstream movements to compensate for downstream drift [19]. Consequently, the “drift paradox” (DP) was developed to articulate the apparent contradictory pattern in which drifting stream organisms that are displaced, sometimes great distances downstream during early life stages, can maintain upstream populations [20]. Since its inception, much of the work on the DP has focused on aquatic invertebrates [19, 21] or model simulations [22, 23]. Proposed resolutions to the DP include the process of density dependence, random directional dispersal at the adult stage, and at least partial retention of larvae at upstream sites [24]. Recently, application of the DP has spanned beyond aquatic invertebrates as linkages between the DP and dispersal of a guild of stream fishes known as “pelagic-broadcast spawning” (PBS) fishes were established [25, 26].

Fishes belonging to the PBS reproductive guild spawn nearly neutrally buoyant eggs within the water column, which then develop as they are swept downstream [27–29]. Model simulations predict that eggs and developing ova can be transported long distances downstream, ranging 131–147 km and 468–592 km [30, 31], depending on stream size and habitat heterogeneity [32]. The concept of retention of eggs and ova at upstream locations for some PBS fishes has spurred much debate [33–39] and there is a general paucity of information on the movements of adult PBS fishes based on their diminutive size, and challenges associated with tagging and tracking individuals over broad spatial extents and in large rivers [15, 40]. Existing studies suggest upstream movement by PBS fishes occurs [25, 41] and is altered by stream fragmentation [42, 43]. However, evidence of biased upstream movement by PBS fishes, as predicted by the CCH, is lacking and additional research on the movement of PBS fishes is necessary, particularly the adult life stage when biased upstream movement is hypothesized to occur (e.g., [30]). Pelagic-broadcast spawning fishes have a unique reproductive mode (particularly in freshwater; [44]), and future research should focus on how this life history strategy relates to movement ecology and conservation biology [40].

Conservation of PBS fishes requires that movement information be integrated into conservation and spatial planning, but a necessary first step is that movement attributes be measured and framed in the context of prevailing theories [6]. Declines in populations of PBS fishes have been shown in fragmented streams, suggesting that a critical fragment length that does not restrict movement may play a role in recruitment success [36, 40, 45]. Cyprinids in the genus *Macrhybopsis* would benefit from conservation to prevent widespread extirpations, but the mechanisms by which known causes of decline operate are unknown despite indirect references to the CCH [40, 46, 47]. As with other PBS fishes, *Macrhybopsis* spp. eggs are transparent, non-adhesive, and become semi-buoyant in water, therefore ova are hypothesized to be subjected to long-distance downstream dispersal [27, 28, 31]. *Macrhybopsis* therefore represent an ideal group for testing the applicability of the RMP versus the CCH. In particular, prairie chub (*Macrhybopsis australis*) was designated as a PBS species in recent research [48], has egg characteristics consistent with other PBS fishes [40], and is commonly included within the assemblage of PBS fishes in the Great Plains [48, 49].

Prairie chub is a short-lived fish endemic to the Red River basin of Texas and Oklahoma, USA with a reproductive season spanning April through September [48]. Other life history characteristics include a diet that consists primarily of aquatic macroinvertebrates, a lifespan of 2 years [48] and a maximum size of 70 mm total length (TL, [50]). Prairie chub is a species in need of conservation in both Texas and Oklahoma, was listed as “Vulnerable” by Jelks et al. [51], “Threatened” in Texas, and is currently under consideration for listing under the U.S. Endangered Species Act [52]. The species is susceptible to local extirpations, especially in fragmented reaches isolated by dams (e.g. extirpation of prairie chub above Lake Altus; [53]) and subject to stream dewatering in the Red River basin [40, 45, 50]. Furthermore, potential for increased drought frequency and anthropogenic water withdrawals may have a synergistic and negative effect on prairie chub by drying habitats [54, 55]. However, drought is a natural phenomenon in the southern Great Plains that has shaped adaptations by fishes [56], including source-sink population dynamics connected by movement along river corridors [57]. Consequently, movement is likely a critical aspect of prairie chub persistence, but little is known about movement patterns for the species outside of inference gained from spatial patterns in occurrence [48].

**Fig. 1:**
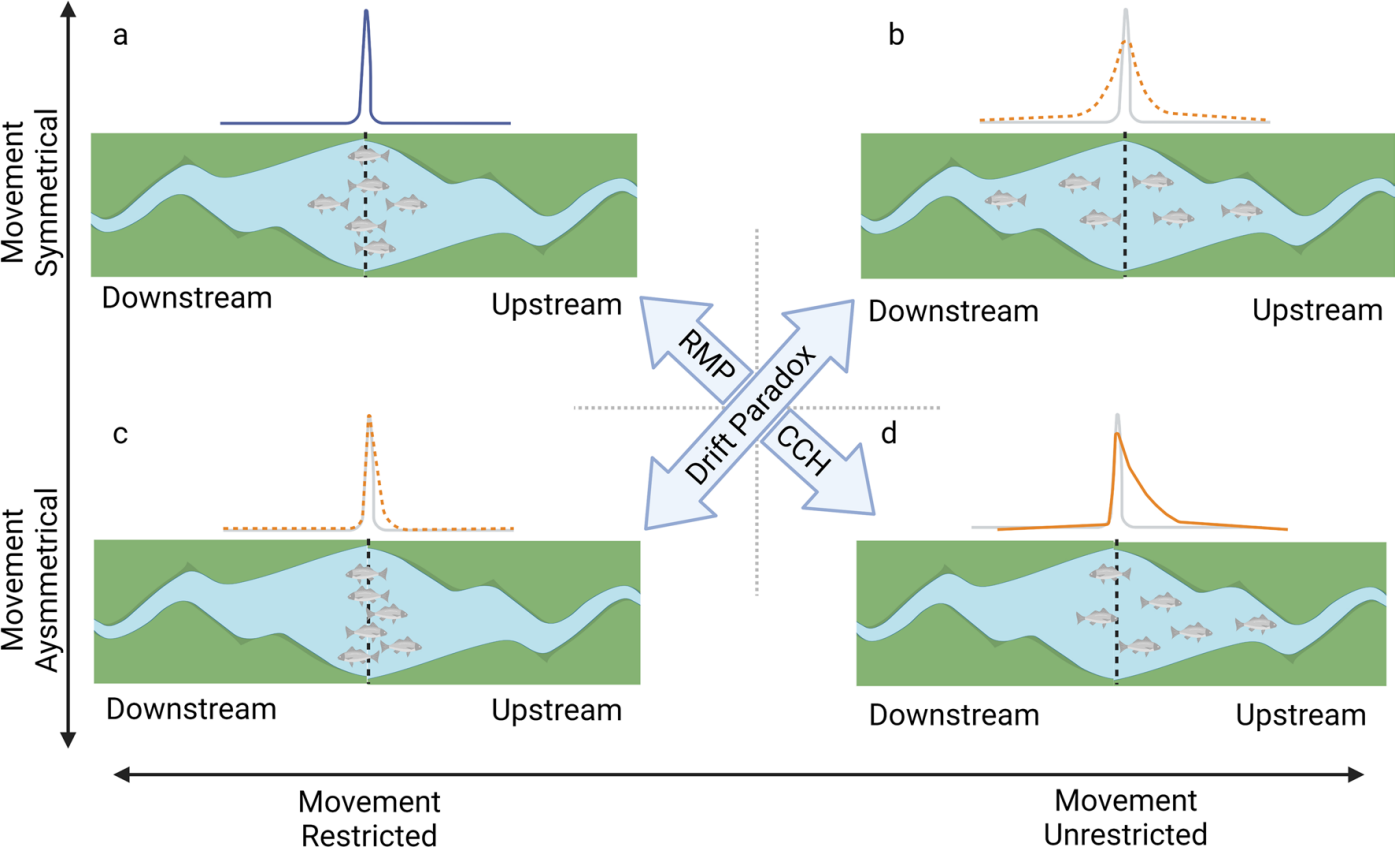
Conceptual diagram showing competing theories for prairie chub movement. The restricted movement paradigm (RMP) posits that stream fish are largely sedentary and do not move far from their tagging location (**a**), while the colonization cycle hypothesis (CCH) posits that if downstream drift occurs during early life stages, then upstream bias in movement must occur at adult life stages (**d**). The drift paradox (DP) describes the situation in which upstream populations persist in spite of little evidence of upstream bias in movement (**b**, **c**). Our first hypothesis (H1) was that prairie chub would move more than the RMP predicts, whereas our second hypothesis (H2) was that prairie chub movement was biased in an upstream direction. Acceptance of both hypotheses would be consistent with the CCH (**d**), whereas rejection of both hypotheses would be consistent with the RMP (**a**). Acceptance of one hypothesis but not the other results in a paradoxical situation in which upstream movement does not complete the colonization cycle (**b**) or the upstream bias is greater than the RMP would predict (**c**)

The purpose of this study was to test the applicability of the RMP versus the CCH and DP in describing the movement ecology of prairie chub. We hypothesized that prairie chub movement would be greater than expected under the RMP (H1) because of anecdotal evidence of long-range movements by prairie chub [48] and empirical evidence of such movements by other Great Plains PBS fishes [26, 41]. We also hypothesized that movement would be upstream biased (H2) consistent with CCH as adult fish move upstream to compensate for downstream drift of eggs and larvae [27, 28, 31]. We concluded that support for the RMP would exist if H1 and H2 were rejected (Fig. 1a), while support for the CCH would exist if both H1 and H2 were supported (Fig. 1d). Support for one hypothesis but not the other is consistent with a paradoxical pattern in which upstream movement to compensate for downstream drift is not evident, and thus evokes the DP (Fig. 1b, c). We then used the results of these hypothesis tests to inform conservation by projecting prairie chub movement across the study area to illustrate the scale of movement by the species in a spatially-explicit manner.

## Methods

### Study area

**Fig. 2:**
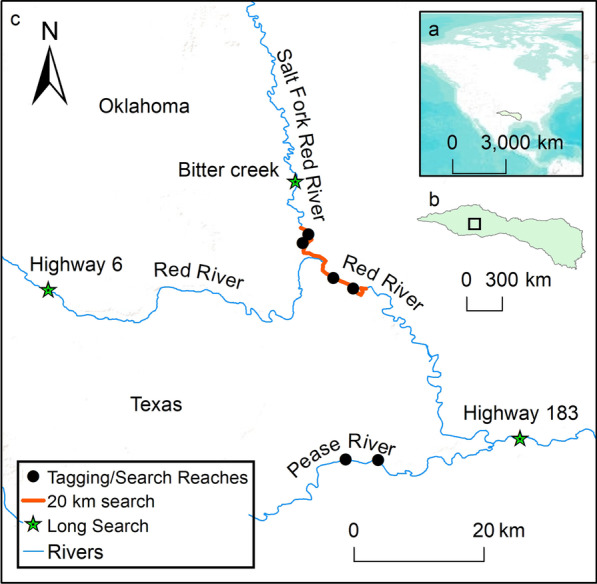
Sample sites located within North America (panel **a**) in the Red River basin (panel **b**) that were used in our mark-recapture analyses of prairie chub movement. Tagging and searching sites were located on the Salt Fork Red River, the Red River, and the Pease River (panel **c**). The long searches were completed August 8–9, 2020 to look for relatively long-distance movers outside of our tagging and regular search sites. The 20-km search was completed August 10–12, 2020 to systematically look for tagged prairie chub above and below 4 of 6 sites along the Salt Fork Red River and the Red River (August 10–12, 2020). NHDplus flowline was used for the rivers [58]

We studied the movement ecology of prairie chub in the upper Red River basin located in the Central Lowlands physiographic province of Oklahoma and Texas, USA. This semi-arid region receives, on average, 82 cm of precipitation annually, and has a mean annual air and water temperature of 18.0 °C and 19.3 °C, respectively [58]. The Red River is characterized by a gently sloping floodplain with a sandy bed that may be up to a kilometer wide and experiences unpredictable seasonal flow variability [58]. Land use in this area is primarily agricultural, with 80–90% being used for rangeland and cropland that is both irrigated and unirrigated [58]. We focused on six sites: two on the Red River (6th order, [59]), two on the Salt Fork Red River (5th order), and two on the Pease River (5th order; Fig. 2). We selected these streams because they are inhabited by prairie chub but occur upstream of a zone of hybridization with closely related shoal chub (*M. hyostoma*; [60]). Daily discharge was monitored throughout our study period by the United States Geological Survey ([61]; Additional File 1) on the Pease River at Vernon, Texas (USGS gage 07308200), the Salt Fork Red River at Elmer, Oklahoma (USGS gage 07301110), and the Red River proper at Burkburnett, Texas (USGS gage 07308500).

### Survival and tag retention

We first assessed tag retention and survival to determine the minimum total length (mm) of prairie chub that could be tagged in the movement experiment. We conducted two 24-h tagging trials using a 68-L perforated tub placed in the Red River at the state highway 283 access point. Fish were individually netted and tagged by injecting visible implant elastomer (VIE) into the muscle tissue just under the scales with a single 2-mm fluorescent elastomer mark (Northwest Marine Technology Inc.) using a 0.3‐mL syringe and a 27‐gauge, 12‐mm long needle. Colored elastomer was injected as the needle was withdrawn, creating a streak until the bevel of the needle reached the injection point [62]. The first trial took place on July 23, 2019 and included one treatment group (single VIE tag, n = 24) and one control group (untagged, n = 24). The second trial took place on August 5, 2019 and included two treatment groups (single tag, n = 23; double tag, n = 24) and one control group (untagged, n = 23). For each of these trials, we collected prairie chub from the mainstem Red River and pooled all collected fish in a single tub. We then randomly netted fish one at a time from the tub and alternated assignment to control and treatment groups that were housed in separate tubs [63]. Control group fish were handled but not tagged or measured, single tag fish received one dorsal VIE tag at the posterior end of the caudal peduncle and were measured for total length, and double tag fish received two dorsal VIE tags at the posterior end of the caudal peduncle and were measured for total length. At the completion of the 24-h trials, fish were classified as retaining (tag still present on fish body) or shedding (tag not evident) their tag and as alive or dead [64]. Attempts to measure tag retention over longer time periods were unsuccessful due to logistical challenges caused by holding fish in captivity.

We analyzed survival using generalized linear regression in the form of a multiple logistic regression model [64], where survival was a binomial response (0 = dead, 1 = alive), length was a continuous independent variable, and treatment was a categorical factor (control, single tag, double tag). We did not model tag retention because all fish retained their tags during the trials. We used the ‘glm’ function from the ‘stats’ package in R (version 4.0.4) to fit the model and used the length at which survival equaled 0.50 probability as the minimum size fish to tag in the movement experiment.

### Movement experiment

We assessed movement of prairie chub using a mark-recapture experiment with multiple tag and recapture events during late spring through summer (i.e., May–August) of 2019 and 2020. At each of the six locations, we established a 1-km tagging reach buffered upstream and downstream by 1-km search reaches. In the Red River and Salt Fork Red River, the two tagging reaches were distributed so that they were 1-km apart, resulting in a shared search reach in the middle (Additional File 2). Each 1-km tagging reach was divided into five 0.2-km sub-reaches where fish were batch tagged using VIE. We collected fish for tagging from each sub-reach using four 50-m seine hauls (9.1-m by 1.8-m, 1-m shallow-bag, tapering to 0.5-m) repeated three times (i.e., triple-pass). We ultimately elected to mark fish with VIE for the movement study because this method is widely used in fish movement studies, has minimal mortality (including in our own study; see “Results” section), and small fishes tagged with VIE demonstrated no behavioral changes caused by tagging in previous fish movement studies [65–67].

Fish captured during each pass were held together in a 68 L perforated tub (to allow oxygenated stream water to flow through) for batch tagging with VIE. We used a subset of 140 potential unique combinations of VIE colors and body locations to ensure fish could be traced back to the sub-reach and date in which they were tagged (Additional File 2). We recorded the date, VIE color, body location of the tag, and total length (to nearest 1-mm) for each tagged fish and placed them in a second aerated 68-L recovery tub for 2 h prior to release [15]. We recorded global positioning system (GPS) coordinates at the release site (i.e., center of sub-reach) for all fish using a handheld Oregon 700 series GPS (Garmin, Olathe, KA, USA). Fish that were recaptured more than once were treated as independent data points and movement was measured from the last release location.

For both study years, we conducted tagging and recapturing events at 2–6-week intervals in each of the three streams (Table 1). During each visit, we spent two consecutive days tagging fish and then conducted recapture searches across all search and tagging reaches on the third day. This approach resulted in an increasing number of fish tagged throughout the summer and provided opportunity to recapture fish over a wider range of time periods. We made recaptures by conducting 50-m seine hauls across the entire search and tagging reaches (Additional File 2). On August 8–9, 2020, we searched three far distance sites using 50-m seine hauls across a 2-km extent of stream upstream and downstream of the mainstem Red River site (Fig. 2). On August 10–12, 2020, we completed a 20-km (i.e., 400 50-m consecutive seine hauls) long-distance search for recaptures from the Salt Fork Red River to the Red River mainstem across 4 of our 6 study sites (i.e., excluding the Pease River tributary sites; Fig. 2). All fish captured during long distance searches were visually scanned for VIE tags independently by two observers. Recapture efforts targeted habitats most likely to be inhabited by chub [68], including habitats near the stream thalweg where water is deepest and fastest. We recorded date, total length (mm), GPS coordinates, VIE color, and body tagging location for each recapture.

**Table 1:** Sampling dates of prairie chub (*Macrhybopsis australis*) during summers of 2019 and 2020 for the Red River, Salt Fork Red River, and Pease River in the Red River Basin of Texas and Oklahoma, USA showing the number of fish tagged (T) and recaptured (R) and discharge (cubic meters per second)

Summer 2019	River	T(R)	Flow (cms)	Summer 2020	River	T(R)	Flow (cms)
Jun. 5–11	Pease	86(4)	15.12	Jun. 19–22	Pease	332(2)	0.44
Jul. 16–17	Pease	88(0)	1.08	Jul. 7–9	Pease	27(1)	1.16
Jul. 31–Aug. 2	Pease	110(1)	0.24	Jul. 24–26	Pease	11(1)	0.37
Aug. 9–12	Pease	95(2)	0.02	–	–	–	–
Total	Pease	379(7)		Total	Pease	370(4)	
May 15–18	Red	576(3)	302.99	Jun. 16–18	Red	955(13)	5.52
Jun. 25–27	Red	113(6)	61.45	Jul. 4–6	Red	404(19)	5.10
Jul. 19–22	Red	95(2)	27.92	Jul. 21–23	Red	677(12)	2.97
Aug. 3–5	Red	96(1)	14.53	Aug. 12	Red	NA(18)	2.18
Total	Red	880(12)		Total	Red	2036(62)	
Jun. 28–Jul. 1	Salt Fork	59(1)	4.87	Jun. 24–26	Salt Fork	284(4)	1.10
Jul. 23–25	Salt Fork	191(3)	3.23	Jul. 17–19	Salt Fork	404(26)	0.76
Aug. 6–8	Salt Fork	436(20)	2.75	Aug. 4–6	Salt Fork	178(18)	0.31
Aug. 13–15	Salt Fork	541(51)	3.14	Aug. 10–11	Salt Fork	NA(5)	0.27
Total	Salt Fork	1227(75)		Total	Salt Fork	866(53)	
Grand Total	All	2486(94)		Grand Total	All	3272(119)	

The final sampling dates for the Red River and Salt Fork Red River in August 2020 were long distance searches and no fish were tagged

### Restricted movement paradigm

We tested the hypothesis that prairie chub would move further distances than expected under the RMP (H1) using the R package ‘fishmove’ [14]. We first estimated expected movements using the function ‘fishmove’, which generates a double-normal distribution of movement distances at the population level using stream size (stream order; [59]), fish length (total length, mm), fish morphology (aspect ratio of caudal fin; [69]), and time at large (days, d) based on the meta-analysis conducted by Radinger and Wolter [14]. We parameterized the expected movement model with the largest stream order we studied (6th order), the median length (mm TL) of adult individuals we captured during this study (54-mm in 2019; 58-mm in 2020), a caudal fin aspect ratio we estimated from scientific images of prairie chub (1.09; [70]), and the median number of days between mark and recapture for all recaptured fish in our study (8 d in 2019; 18 d in 2020). This function provides an estimate and 95% confidence interval for distances moved by the stationary (sigma-stat) and mobile (sigma-mob) components of the population. Next, we estimated a movement distribution curve from our mark-recapture field data using the function ‘fishmove.estimate’, which fits a double normal distribution to a vector of movement distances observed in the field to generate estimates of distances moved by mobile and stationary components of the population. We then assessed whether the estimate for observed movement fell within the 95% confidence intervals of the expected movement and accepted H1 if the observed movement distance was greater than the upper 95% confidence interval for expected movement. We repeated this test for 2019 and 2020 separately, for net movement, defined as the linear distance (m) between tagging and recapture locations along the stream thalweg, which was measured using the network analyst function in ESRI ArcGIS (ESRI, Redlands, CA). We also used daily movement rate (m/d), defined as net distance moved divided by the number of days between tagging and recapture. We estimated the expected movement rate by changing the time interval in the function ‘fishmove’ from 8 d (2019) or 18 d (2020) to 1 d so that estimates of movement distance were standardized by time.

### Colonization cycle hypothesis

We tested the hypothesis that prairie chub exhibited biased upstream movement expected with the CCH (H2) using frequency histograms and distances/rates moved upstream versus downstream. We tested for skewness, kurtosis, and upstream bias based on 2019 and 2020 recapture data. We tested normality and kurtosis of net movement (m) and daily movement rate (m/d) distributions using D’Agostino’s test for normality [71] and Anscombe Glynn’s test of kurtosis [72] following previous methodologies [15, 73, 74]. We tested our hypothesis that prairie chub net movement (m) and daily movement rate (m/d) was biased in an upstream direction (H2) using a Mann–Whitney U test implemented with the ‘wilcox.test’ function in R [75]. We converted the distances moved upstream versus downstream to absolute values (i.e., instead of representing downstream distances with a negative value) and then tested for differences between the ranks of distances in either direction. All functions were executed in R version 4.0.4 (R Core Team 2021; Additional File 3).

### Conservation implications

We investigated the conservation implications of the results from our hypothesis testing with respect to the scale of prairie chub movement. The mobile component of populations is critical for connecting meta-populations and recolonization of habitat [12, 76], including prairie chub in particular [48]. Consequently, we compared the movement rate (m/d) for the mobile component of the prairie chub population with all other species included in the work by Radinger and Wolter [14]. The list of species made up of several different fish families is included in the R package ‘fishmove’ and is available within the package (‘fishmove:::speciesfishmove’). We estimated the daily (i.e., time = 1 d) movement of the mobile component for each of these species. We then plotted the daily movement rate for the mobile component of the prairie chub population from our study for 2019 and 2020 separately to illustrate the contrast between prairie chub movement and movement by other river fishes analyzed by Radinger and Wolter [14]. Next, we estimated the range (i.e., maximum distance that prairie chub might move) for the mobile component by multiplying the estimated movement rate (m/d) by 123 days, which encompassed the summer season for which we collected movement data (May 1 through August 31). Greater movement might be possible if time periods outside of this season are included, but we did not measure movement for other seasons. We calculated potential ranges for 2019 and 2020 separately and then used the locations of our tagging as source locations to estimate the upstream and downstream ranges of prairie chub. The resulting spatial extent of the prairie chub range may effectively provide a footprint of scale for future conservation and management decisions.

## Results

### Survival and tag retention

**Fig. 3:**
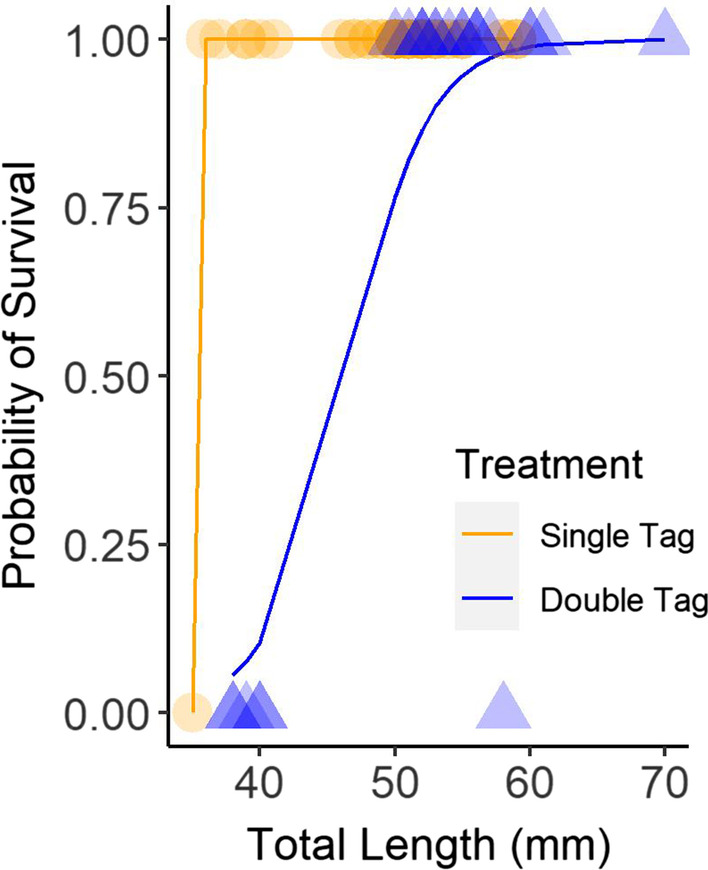
Probability of survival over a 24-h period for prairie chub tagged with a single visible implant elastomer (VIE) tag (orange circles, n = 47) and double VIE tag (bluetriangles, n = 24) as a function of fish total length (1 mm). Lines of corresponding colors show logistic regression model fits for each treatment level and points with darker colors illustrate higher densities of observations

Prairie chub tag retention and survival were generally high. Both trials had a tag retention rate of 100% after 24 h. The control group experienced 100% survival. Survival of the single tag treatment group was 98% [46 of 47], and survival of the double tag treatment group was 75% [18 of 24]. Results from the regression model showed a significant treatment effect (Z = 2.36, *p* = 0.02) and a significant effect of length (Z = 2.66, *p* = 0.01). The only mortality in the single tag treatment group was a fish that was 35-mm TL, the smallest fish in the treatment group. The six mortalities in the double tag treatment group included the five smallest fish (range = 38–40 mm TL) and one fish that was 58-mm TL. The probability of survival exceeded 0.50 for double-tagged fish at 45-mm TL (Fig. 3), and we did not double-tag fish in the movement experiment if they were less than this length.

### Movement experiment

Prairie Chub movement was determined by using mark-recapture methods during four visits to each site during 2019 and three visits to each site during 2020. We tagged 5771 prairie chub during summer 2019 (n = 2499) and 2020 (n = 3272) and recaptured 213 fish across both summer 2019 (n = 94) and summer 2020 (n = 119). The average length of fish recaptured was 54-mm TL (range 42–70) during 2019 and 58-mm TL (range 48–67) during 2020. Fish were at large for an average of 11 d in 2019 (median = 8, range = 1–57) and 17 d in 2020 (median = 18, range = 1–68). A single fish that was tagged in summer 2019 was recaptured during summer 2020. This fish was tagged at the lower site in the Red River on July 21, 2019 and was recaptured at the lower site on the Salt Fork River on July 17, 2020. The prairie chub moved at least 11,745 m upstream over 362 d (movement rate = 32.4 m/d). We removed this observation from our analysis because it represented the only recapture between the two summers. We recaptured two and three individuals that were double recaptured in 2019 and 2020, respectively, which were treated as new recaptures for the purpose of analysis. All movement distributions were leptokurtic, including net distance moved in 2019 (Additional File 4a, kurtosis = 15.3, z = 5.70, *p* value < 0.01) and 2020 (Additional File 4b, kurtosis = 5.57, z = 3.39, *p* value < 0.01) and daily movement rate for 2019 (Additional File 4c, kurtosis = 12.7, z = 5.34, *p* value < 0.01) and 2020 (Additional File 4d, kurtosis = 21.9, z = 6.76, *p* value < 0.01). We recaptured very few tagged fish outside of our usual survey extents. The long-distance searches at state highway 6, state highway 183, and below the confluence of Bitters Creek yielded no recaptured prairie chub. The 20-km long distance search yielded recaptures that were all within the normal tagging and search reaches with the exception of one fish that moved 69 m below the Salt Fork lower search site.

### Restricted movement paradigm

**Fig. 4:**
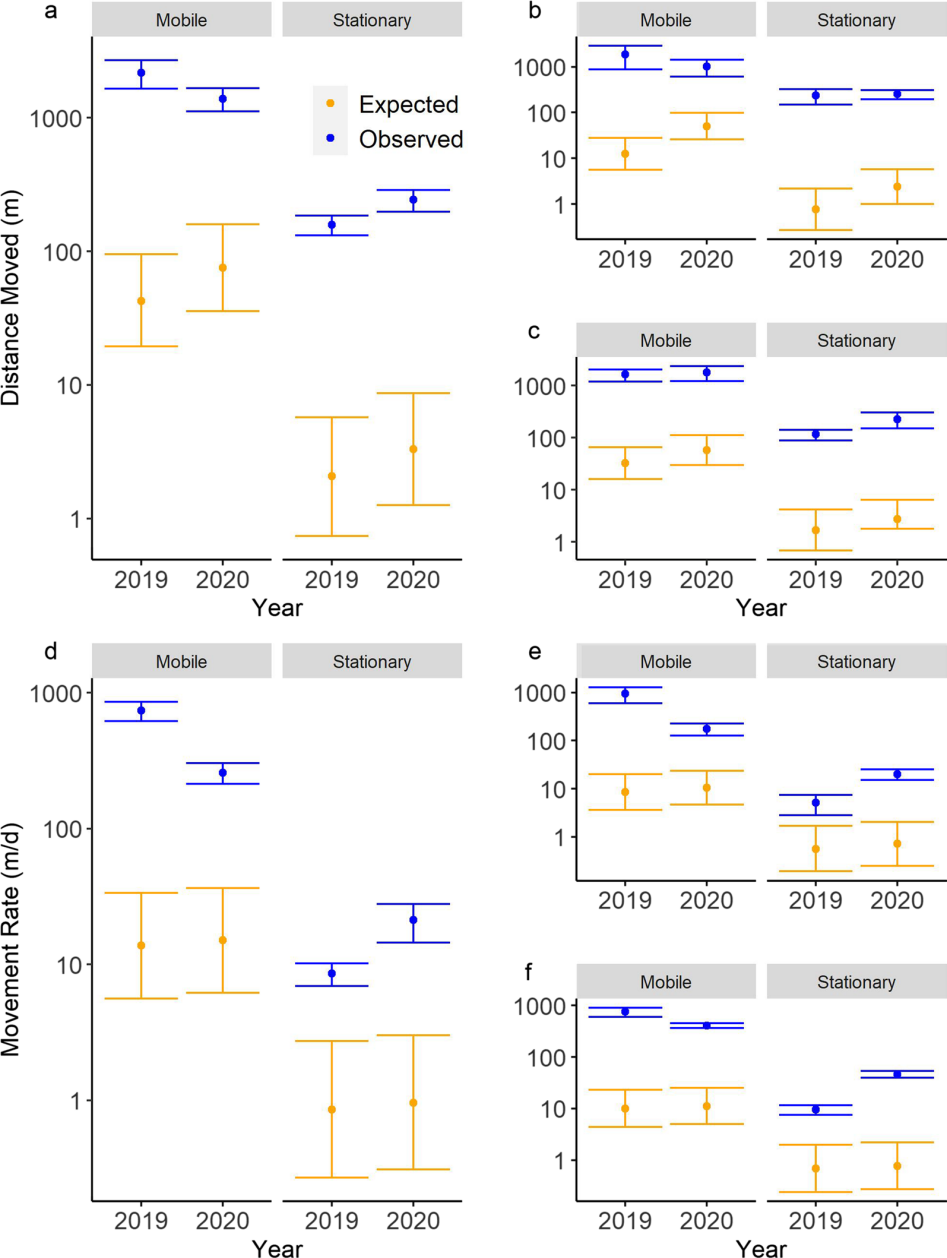
Plot of expected (orange) versus observed (blue) prairie chub (**a**, **b**, **c**) movement distances and (**d**, **e**, **f**) movement rates for mobile (left) and stationary (right) components of the population measured during the summers of 2019 and 2020. The plot has pooled (**a**, **d**; 2019 n = 94, 2020 n = 119) data which contains all sites, the Red River (**b**, **e**; 2019 n = 12, 2020 n = 62) only, and the Salt Fork River (**c**, **f**; 2019 n = 75, 2020 n = 53) only. The Pease River was not included due to low recapture numbers (2019 n = 7, 2020 n = 4). The orange bars around expected movements are upper and lower 95% confidence intervals generated using the ‘fishmove’ function in R. The blue bars around observed movements are upper and lower 95% confidence intervals calculated from the standard errors given from the ‘fishmove.estimate’ function in R

Observed prairie chub movement measured by distance and rate were consistently greater than expected under the RMP. The expected movement distances of the stationary and mobile components differed slightly between 2019 and 2020 (Table 2). Observed movement distances were consistently, and statistically, greater than expected across years and study systems (Fig. 4a–c). Similarly, the expected movement rates for 2019 were not significantly different between 2019 and 2020, but observed movement rates were consistently greater than expected across years and study systems (Fig. 4d–f). The expected share of the stationary component (*p*) for movement distance and rate was 0.67, and the observed values for *p* were close to this value during 2019 (distance = 0.79; rate = 0.60) and 2020 (distance = 0.69; rate = 0.63). Based on these data, we accepted H1 and concluded that prairie chub moved greater distances and at faster rates than expected under the RMP.

**Table 2:** Data from the expected (‘fishmove’) and observed (‘fishmove.estimate’) output from R for prairie chub movement distance and rate in the Red River, Salt Fork Red River, and Pease River in the Red River Basin of Texas and Oklahoma, USA during 2019 and 2020

River	Movement	Movement	2019		2020	
	Metric	Component	Expected	Observed	Expected	Observed
Pooled	Distance	Stationary	2.1 (0.7–5.8)	158.6 (131.7–185.6)	3.3 (1.26–8.7)	242.8 (197.8–287.8)
Pooled	Distance	Mobile	42.5 (19.5–94.9)	2169 (1647–2691)	75.6 (35.8–159.3)	1391 (1115–1668)
Red	Distance	Stationary	0.8 (0.3–2.2)	235.9 (147.5–324.4)	2.4 (1.0–5.7)	254 (195.2–312.8)
Red	Distance	Mobile	12.6 (5.6–28.1)	1879.6 (878.6–2881)	50.2 (25.7–98.4)	1029 (617.4–1441)
Salt Fork	Distance	Stationary	1.7 (0.7–4.2)	116 (89.1–142.9)	2.7 (1.8–6.4)	227 (150.6–303.4)
Salt Fork	Distance	Mobile	32.3 (16.0–65.4)	1629 (1202–2056)	57.7 (30–111)	1790 (1234–2346)
Pooled	Rate	Stationary	0.9 (0.3–2.7)	8.6 (6.9–10.2)	1.0 (0.3–3.0)	21.2 (14.5–27.9)
Pooled	Rate	Mobile	13.7 (5.6–33.7)	739.8 (619.8–859.8)	15.0 (6.2–36.5)	258.2 (212.9–303.6)
Red	Rate	Stationary	0.6 (0.1–1.7)	5.1 (2.8–7.3)	0.7 (0.25–2.1)	20.1 (15.1–25.1)
Red	Rate	Mobile	8.5 (3.6–19.9)	947 (597.2–1297)	10.5 (4.6–23.8)	176.4 (127.1–225.6)
Salt Fork	Rate	Stationary	0.7 (0.2–2.0)	9.5 (7.6–11.5)	0.8 (0.3–2.2)	46.4 (39.7–53.1)
Salt Fork	Rate	Mobile	10.1 (4.4–23.0)	755.5 (602.7–908.2)	11.2 (5.0–25.2)	410.1 (364.8–455.5)

The pooled data (2019 n = 94 recaptures, 2020 n = 119) represents the Pease (2019 n = 7, 2020 n = 4), Red (2019 n = 12, 2020 n = 62), and Salt Fork (2019 n = 75, 2020 n = 53) rivers combined. We did not analyze the Pease River alone due to insufficient sample size. Distance (meters), Rate (meters per day). Values are fitted means (95% confidence intervals)

### Colonization cycle hypothesis

**Fig. 5:**
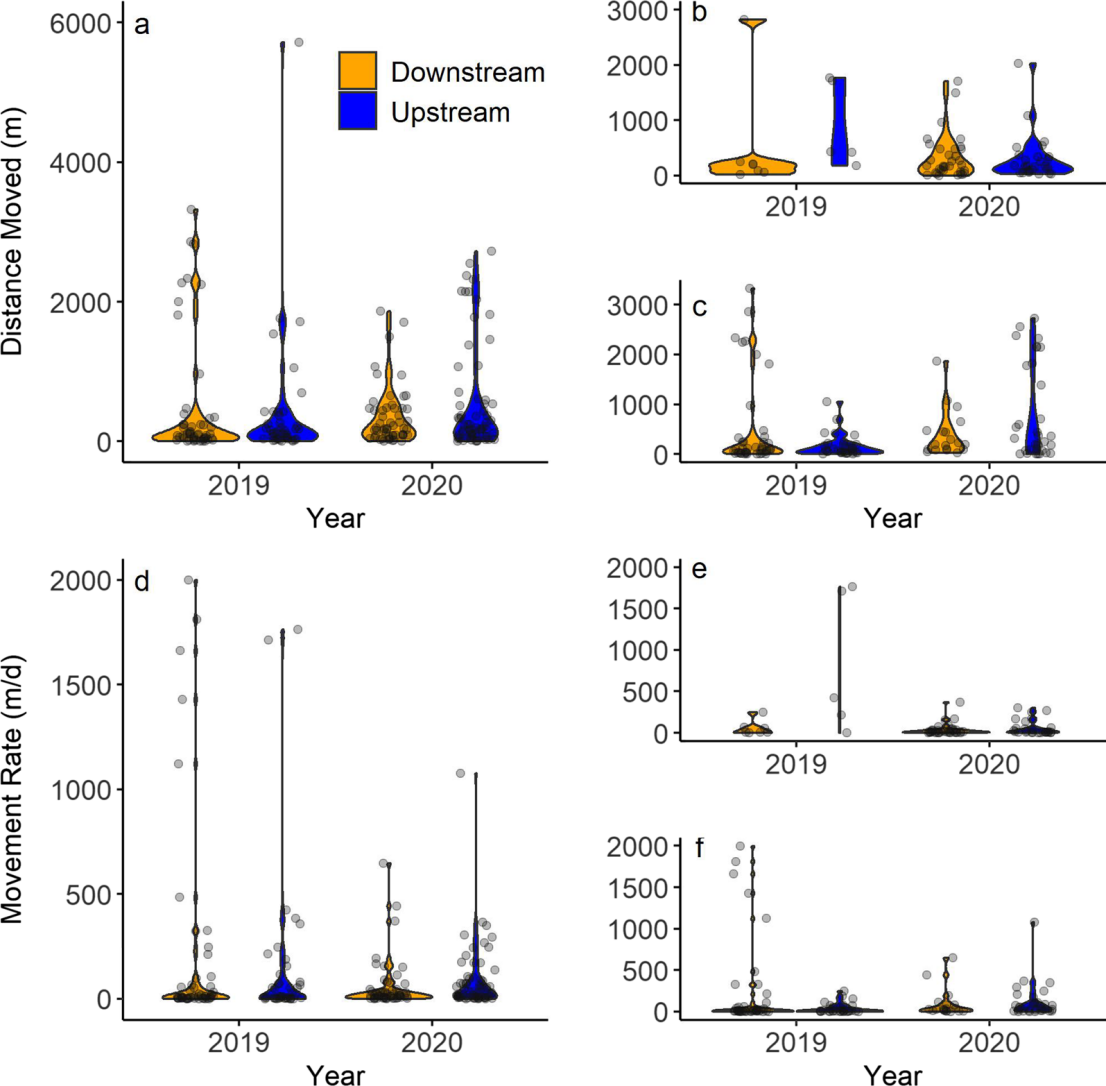
Violin plots comparing (**a**, **b**, **c**) absolute distance moved (m) and (**d**, **e**, **f**) movement rate (m/d) in downstream (orange) and upstream (blue) directions for prairie chub recaptured across all sites (**a**, **d**; 2019 n = 42 upstream and n = 51 downstream, 2020 n = 64 upstream and n = 54 downstream), the Red River only (**b**, **e**; 2019 n = 5 upstream and n = 7 downstream, 2020 n = 34 upstream and n = 28 downstream), and the Salt Fork Red River only (**c**, **f**; 2019 n = 32 upstream and n = 42 downstream, 2020 n = 33 upstream and n = 19 downstream) during the summers of 2019 and 2020. The Pease River was not included due to low recapture numbers (2019 n = 7, 2020 n = 4). The width of each violin plot denotes data density

There was limited evidence of upstream bias in adult (i.e., ages 1 and 2) prairie chub movement during the summers of 2019 and 2020. We found no difference in the distances or rates moved upstream versus downstream for 2019 or for 2020 based on Mann–Whitney U tests of the pooled data, Red River only, or Salt Fork Red River only (Table 3). Violin plots illustrated consistent distributions of distances moved upstream and downstream (Fig. 5a–c) as well as consistent rates moved upstream versus downstream (Fig. 5d–f). Frequency distributions plotted in continuous bins including both upstream and downstream (Additional File 4) were skewed positively for distance in 2019 and 2020, but rates were negatively skewed in 2019 and positively skewed in 2020 (Table 3). Based on these data, there was limited support for H2 and we concluded that prairie chub did not exhibit highly biased upstream movement during summer months of their adult life stage as predicted by the CCH.

**Table 3:** Results of tests applied to upstream versus downstream movement distances and rates of prairie chub in the Red River, Salt Fork Red River, and Pease River in the Red River Basin of Texas and Oklahoma, USA during 2019 and 2020

River System	Movement Measurement	2019						2020					
		US/DS	MWU Test Statistic (W)	*p* Value	Skew	Test Statistic (Z)	*p* Value	US/DS	MWU Test Statistic (W)	*p* Value	Skew	Test Statistic (Z)	*p* Value
Pooled	Distance	42/51	1035	0.78	0.97	3.58	< 0.01	64/54	1601	0.49	1.19	4.61	< 0.01
Red	Distance	5/7	8	0.15				34/28	482	0.938			
Salt Fork	Distance	32/42	732	0.52				33/19	283	0.57			
Pooled	Rate	42/51	1063	0.95	− 1.00	− 3.67	< 0.01	64/54	1428	0.11	1.88	6.22	< 0.01
Red	Rate	5/7	7	0.11				34/28	414	0.38			
Salt Fork	Rate	32/42	759	0.35				33/19	269	0.41			

The number of fish moving upstream (US) versus downstream (DS) are given for the pooled data (Red River, Salt Fork Red River, and Pease River), Red River only, and Salt Fork Red River only. We did not analyze the Pease River individually because of insufficient sample size. Test statistics and *p* values are given for Mann–Whitney U (MWU) tests of ranked movement distances and rates for upstream versus downstream (sample sizes give in US/DS), and skewness values, test statistics, and *p* values are given for frequency distributions of pooled data shown in Additional File 4

### Conservation implications

**Fig. 6:**
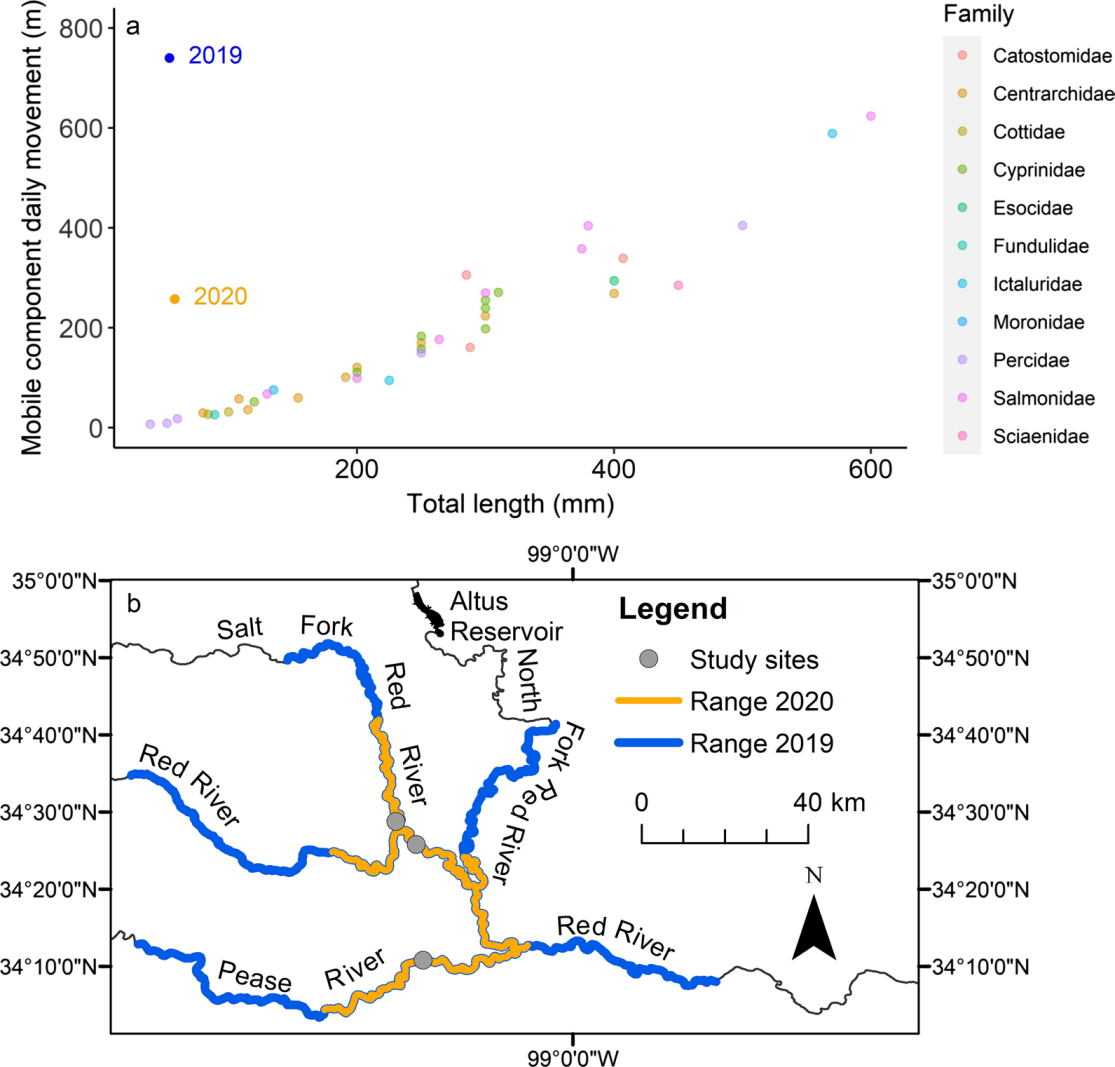
**a** Prairie chub mobile component movement during one day (i.e., daily movement rate) for 2019 (blue) and 2020 (orange) compared with 40 other species in the figure included in the ‘fishmove’ package in R. Prairie chub are the only pelagic-broadcast spawner shown and was not included in the Radinger and Wolter [14] analysis. The x-axis shows mean fish total length (mm) and the y-axis is the distance moved by the mobile component of populations; symbols for fishes from ‘fishmove’ are shown by taxonomic family with prairie chub being part of the family Cyprinidae. **b** Movement range (i.e., maximum distance possible during a single summer) for the mobile component of the prairie chub population for 2019 when flows were higher (blue) compared to 2020 when flows were lower (orange). Movement range is measured from the locations where fish were tagged (gray points) and does not include other locations where the species is known to occur

Comparison of prairie chub with the 40 species included in the package ‘fishmove’ illustrated that the mobile component of the prairie chub population moved at a much higher rate (m/d) compared with other fishes, including that of other cyprinids [14]. Given their size, prairie chub movement rate was 28-times (2020) to 82-times (2019) faster than expected based on data from 40 other river fishes (Fig. 6a). Consequently, prairie chub conservation and management will likely differ from the typical riverine fish of its size. When extrapolated across the 123 days of the summer season including the months of May (31 days), June, (30 days), July (31 days), and August (31 days), the potential range of prairie chub was 31.7 km during 2020 and 91.0 km during 2019. Using only the three tagging locations as sources of movement, the mobile component range during 2020 connected the Red River mainstem, Salt Fork Red River, North Fork Red River, and Pease River, and an even larger range during 2019 included a greater extent of all these streams (Fig. 6b).

## Discussion

Our study synthesizes and integrates prairie chub movement into prevailing movement theories. Ruppel et al. [48] recently inferred seasonal movement of prairie chub based on occurrence of age groups along upstream to downstream gradients in the Pease and North Fork Wichita rivers in Texas. Though the authors concluded that prairie chub are capable of long-distance upstream movements, no quantitative assessment of movement was provided. Wilde [77] used VIE tagging and mark-recapture to track movement of closely related peppered chub (*Macrhybopsis tetranema*) in the Canadian River of Texas, the presumed last remaining population for that species [47]. However, because of limited recaptures, movement rate could only be estimated when combined with plains minnow (*Hybognathus placitus*) and Arkansas River shiner (*Notropis girardi*) to yield a rate of 370 m/d [77]. Compared with this estimate, our empirical measures of prairie chub movement rate for the mobile component were twice as fast for 2019 (i.e., 740 m/d) and slightly slower for 2020 (i.e., 258 m/d). These results collectively point to prairie chub and closely related peppered chub moving further than expected under the RMP. However, we did not find bias in summer upstream movement as would be expected under the CCH and as previously hypothesized for species such as peppered chub [78]. Instead, we found that summer movements were strongly leptokurtic and largely symmetrical in terms of upstream versus downstream distances. This finding is consistent with modeling simulations from Speirs and Gurney [22] and empirical results from a movement study of flathead chub (*Platygobio gracilis*) in a Colorado stream [43]. Speirs and Gurney [22] simulated and modelled organism persistence in environments characterized by advection (e.g., streams and rivers) and determined that the likely mechanism for upstream population persistence is diffusive dispersal rather than simply biased upstream movements. Increased dispersal, regardless of directionality, returns an ample number of individuals upstream such that populations persist at their natal upstream location. Flathead chub showed upstream bias in reproductive readiness but not movement (April–October), although an artificial barrier placed on the stream blocked upstream movement. Later evidence suggested that flathead chub produce non-adhesive eggs that are potentially displaced long distances downstream, which alludes to necessary upstream movement as described by the CCH [79]. Thus, for at least flathead chub in Colorado and prairie chub in our study system, neither the RMP nor the CCH fully describes summer movement dynamics by adult fish. These findings highlight an existing drift paradox for Great Plains fishes that will only be resolved through greater focus on movement ecology [25, 26, 48]. For example, our research demonstrates that biased upstream movement does not occur for adults during summer months, but future research could uncover upstream bias by focusing on earlier life stages or other seasons.

Resolving the drift paradox for Great Plains fishes will require a deeper understanding of their reproductive biology and movement ecology. The need for ova to drift downstream in order for PBS propagules to survive was first proposed by Moore [29] for Arkansas River shiner and similar characteristics were later noted for peppered chub [27]. Later observations of spawning by PBS fishes held in captivity, including speckled chub (*M. aestivalis*) and sickelfin chub (*M. meeki*), remain some of the most detailed accounts of Great Plains PBS reproductive ecology [28, 31], although additional basic research on the spawning mode of prairie chub could be further studied to understand drift dynamics [40]. Properties of eggs documented during captive spawning led to the use of egg surrogates, termed passive drifting particles (PDPs), in experiments testing characteristics of drift, displacement, and retention [30, 32, 33, 35]. These works have spurred considerable debate in the literature, a debate that greater information on movements could help to resolve. For example, Medley et al. [33] modelled drift and retention of PDPs that simulated Pecos bluntnose shiner (*Notropis simus pecosensis*) eggs in the middle Pecos River and concluded that retention was high enough in the upstream section of river that drift into the lower, degraded reach posed little threat to the species. Thus, retention of ova and larval fishes may maintain populations upstream even in the presence of downstream drift [80, 81]. Zymonas and Propst [34] reanalyzed the PDP data presented by Medley et al. [33] and suggested that drift into the lower, degraded reach was in fact likely. Chase et al. [41] used otolith microchemistry of adult Pecos bluntnose shiner to show that 82% of specimens caught in the upper reach were originally hatched in the lower or middle reaches and moved to the upper reach while the remaining 18% of individuals hatched in the upper reach and remained residents. This example highlights that retention of at least some ova at upstream locations is possible, but the retained portion is small relative to the portion of the population that might be transported downstream. In the context of prairie chub, collection of a limited number of age-0 individuals at upstream sites by Ruppel et al. [48] suggests that at least a portion of the population is retained upstream, but dominance of age-0 fish downstream is consistent with either downstream displacement during drift or greater recruitment at downstream locations [41, 80, 81]. As a second example, drift rates for PDPs in the North Canadian and Canadian rivers in Oklahoma, two systems comparable to the Red River, ranged 0.07–0.55 m/s depending on discharge and channel geomorphology [32]. These rates equate to 6–47 km/d and over a 3-day period (i.e., presumed larval development timing; [31]) could result in downstream distances ranging 18–143 km. If consistent drift distances occur in the Red River system, our data suggest the mobile component of the prairie chub population could move comparable distances (i.e., 32–91 km) within in a single summer. The conservation of PBS fishes will ultimately require additional research on reproductive life history [40]. Our results provide previously unquantified aspects of adult movement ecology and the appropriate scale of habitat conservation for an imperiled and presumed member of this reproductive guild.

Prairie chub and other ecologically similar species may benefit from management activities that maintain flow and habitat connectivity. Dudley and Platania [30] demonstrated the combined threats that flow alteration and habitat fragmentation pose to PBS fishes, particularly downstream transport of ova into reservoirs where survival and recruitment are presumed limited. Results from several studies in the Great Plains show local extirpations of PBS fishes in truncated stream fragments [30, 32, 36, 48], though the mechanisms are debated. Hoagstrom [37] suggested there was a lack of evidence that fragmentation alone had contributed to PBS fish extirpation, but Wilde and Urbanczyk [38] provided examples of extirpations that occurred upstream of large barriers where interrupted dispersal was a likely explanation. In fact, prairie chub extirpation from the North Fork Red River upstream of Altus Reservoir was attributed to habitat fragmentation [53]. More recently, studies have documented the loss of PBS fishes, including *Macrhybopsis* spp., from fragmented streams that either suffer the effects of extreme drought (e.g., (55, 82)) or the long-term effects of water depletion, extraction, or diversion (e.g. irrigation; [49]). Extreme drought events are known to suppress or thwart recruitment of PBS fishes [83, 84], and under scenarios of local extirpation, populations can only persist when recolonization is possible. Mollenhauer et al. [85] assessed broad-scale occurrence and detection patterns for prairie chub and found detection was lower during extended dry periods compared to wet periods, however, occurrence was only marginally different. We found evidence of greater dispersal in 2019 when flows were higher compared to 2020, which may relate to increased overall detection (i.e., more sites with fish). Further hypothesis testing is needed to completely understand the linkage between flow and dispersal, but positive correlations between flow and dispersal are apparent in other fish populations [15, 76]. Prior to our study, the distances PBS fishes such as prairie chub were able to move to (re)colonize river segments was largely unknown outside of qualitative descriptions of “long distances” [48]. Our work provides empirical evidence for high dispersal and potential movement ranges that far exceed those expected for the average river fish. The validity of this finding is further supported from documented movement by plains killifish (*Fundulus zebrinus*) studied in the same system using the same methods as our study of prairie chub. In particular, plains killifish movement shows strong consistency with the RMP while prairie chub movement does not [16].

Although we provide insight into movements by prairie chub across two summers, our study has a several limitations and continued research may improve understanding of the movement ecology of this species. We tagged prairie chub fish during the known spawning season (April–September; [48]) when gravid females were present. However, it is possible that movement is hindered during this time because fish are contributing energy to reproduction or risk avoidance from predators [64], and movement might have occurred earlier in the year. Furthermore, our study was limited to tracking movements of adult individuals ranging 45–70 mm TL. It is possible that there is an upstream bias in movement among smaller individuals that were too small to tag with VIE (e.g., [25]) as demonstrated by PBS species Pecos bluntnose shiner [41]. We also suggest that basic life history research on ova characteristics of prairie chub and other suspected PBS fishes be done to compliment previous studies [40, 48]. Future studies gathering individual information across a broader range of fish sizes, ages, and seasons would address these limitations of our study. The use of p-chips or other tagging methods to collect individual movement data for diminutive fishes as small as 20 mm standard length [86] would be a good strategy. Another limitation to our study was sample size, though our recapture rate (i.e., 3.8%) was similar to other studies that used VIE on freshwater PBS fishes. Platania et al. [26] tagged 11,500 Rio Grande silvery minnow (*Hybognathus amarus*) and had a 0.6% recapture rate, while Wilde [77] tagged three PBS species with recapture rates varying from 1.4% for Arkansas River shiner, 0.8% for plains minnow, and 8.7% for peppered chub. However, we argue that recapture number is more important than percentage (e.g., 50% recapture of 6 individuals would not give enough statistical power to analyze an entire population) and our numbers of recaptured fish matched or exceeded previous movement studies of fishes [15, 26]. However, we also point out that failure to recapture all or a majority of the tagged individuals, even between years, could be due to movement outside our search areas, predation or other mortality (i.e. two year life span), loss of tags, or non-detection because of imperfect sampling methods. One of the benefits of our study design over previous mark-recapture studies of PBS fishes (e.g., [77]) is that we searched evenly upstream and downstream instead of biasing searches in upstream locations. Albanese et al. [87] suggested that when designing a mark-recapture study, it is important to search equal distances upstream and downstream to reduce bias. Finally, our basis of comparison with the RMP was a meta-analysis that included few small-bodied fishes in large rivers (as highlighted by Archdeacon et al. [88]) and studies such as ours that address this paucity will ultimately improve empirical tests of movement theories.

From a conservation perspective, knowledge that fractions of prairie chub populations make long-distance movements can inform decisions related to maintaining habitat connectivity and flow-based habitat integrity. Historical range reduction of prairie chub has occurred in fragmented habitats where access is now blocked by reservoir dams [48, 53]. The largest of these habitats is located far upstream within the North Fork of the Red River and if not blocked by a dam would be within the swimming range of the mobile component of the population in the lower North Fork Red River based on our results. We hypothesize that this area historically required meta-population connectivity over broad spatial extents, perhaps because temporally variable desiccation disturbances are more effective at causing population declines in smaller streams [82]. Consequently, consideration of species occurrences (or loss of occurrence after habitat alteration) should account for the fact that movement might be a critical aspect of niche definition [90]. This is the basis for greater integration of movement ecology into conservation biology [1]. One concept in particular that might be useful for organisms such as prairie chub that occupy highly variable habitats is the idea that movement is not classified as migratory or resident, but might instead be nomadic. Teitelbaum and Mueller [91] reviewed nomadic movements by animals and concluded that ecosystems with high inter-annual environmental variability should select for nomadic movements, and within these ecosystems variation in movement among individuals could manifest as mixes of stationary and randomly mobile behaviors as we observed for prairie chub (especially with regard to upstream vs downstream directions). Runge et al. [3] further suggested that conservation of species that display nomadic movements can be challenging because their geographic distributions are viewed and managed as static when in fact, they may be temporally dynamic and strongly tied to movement. Consequently, conservation-focused management actions for prairie chub would benefit from consideration of maintaining connectivity on the order of 10^3^–10^5^ m [7] and across a range of habitats including mainstem and tributary ecosystems [92].

## Conclusions

We employed a mark-recapture study at an unprecedented spatial extent (i.e., recurrent continuous searches five km in length) and found that prairie chub movement was at least an order of magnitude greater than expected under the prevailing stream fish movement ecology paradigm (i.e., the RMP; [10, 15]). This finding supports earlier observations and hypotheses related to fishes such as prairie chub engaging in long-distance movements over relatively short temporal extents (i.e., within a single summer; [48, 77, 89]). However, we found only limited evidence for the presumed upstream bias in movement frequently ascribed to PBS fishes [28]. Specifically, distances moved upstream versus downstream did not differ despite positive skewness in most movement distributions. These seemingly contradictory patterns in which fish move great distances but not in a synchronized upstream fashion present a paradoxical situation in which upstream populations of prairie chub persist despite presumed downstream drift at early life stages (i.e., the DP; [19–21]). The resolution to this paradox is likely related to two features of their ecology. First, downstream displacement of ova is unlikely to be uniform and therefore some upstream retention is plausible in this system [41, 80, 81]. Second, previous modelling and real-world observations in stream systems show that increased dispersal (even in the absence of upstream biased dispersal) is sufficient to facilitate population persistence when critical lengths of habitat persist [22]. These observations advance an emerging narrative in which PBS fishes require minimum fragment lengths [45], are subject to increased downstream displacement under modified flows [30], and experience some upstream retention [41, 44, 80, 81], but do not show strongly biased upstream movement [26, 42, 43].

## Supplementary Information

**Additional file 1**. Hydrographs for the Red River (U.S. Geological Survey gage # 07308500), Salt Fork Red River (USGS gage # 07301110), and Pease River (USGS gage # 07308200) indicating daily discharge from May through September of (a) 2019 and (b) 2020. Lines are colored by river and similarly colored points on lines represent sampling dates. Note the y-axis is shown on a log_10_ scale.

**Additional file 2**. Conceptual diagram for a visible implant elastomer (VIE) mark-recapture study to assess movement of Prairie Chub. On each of three tributaries, we established a study segment that was 5 km in length. The study segments comprised 5, 1-km reaches. Every other reach was a search or tagging reach, in which fishes were either only searched for (i.e., search reach) or tagged and searched for (i.e., tag reach). Each tag reach comprised 5, 0.2-km sub-reaches, and within these sub-reaches fish were batch marked with sub-reach-specific body locations. This allowed greater resolution of fish movement during recaptures. During each tagging trip, a new color of VIE was used so that time at large (days) for recaptured individuals could be calculated and associated with a particular date. Picture of VIE tags are from Northwest Marine Technology Inc. and the black and white chub picture is from Everman [82]. There are examples of tagged prairie chub with various VIE colors.

**Additional file 3**. This file contains the R code used in this manuscript and data files used in analysis.

**Additional file 4**. Frequency histograms of prairie chub movement distance (m) and rate (m/d) for 2019 (a, c; n = 94) and 2020 (b, d; n = 119). Colors correspond with fish recaptured in the Pease River (green), Red River (orange), and Salt Fork Red River (blue) and are shown as stacked bars. Negative values represent downstream movement and positive values represent upstream movement. Distance moved is shown using 100 m bins and movement rate is shown using 50 m bins.

## Data Availability

All data generated and analyzed from this study are included in the published article and supplementary materials.

## Abbreviations

RMP: Restricted movement paradigm
CCH: Colonization cycle hypothesis
DP: Drift paradox
PBS: Pelagic-broadcast spawning
TL: Total length
d: Days
mm: Millimeters
cm: Centimeters
VIE: Visible implant elastomer
