# Supplementary figures and images for: Paradigm versus paradox on the prairie: testing competing stream fish movement frameworks using an imperiled Great Plains minnow
Source: Mov Ecol. 2022 Feb 22;10:8. doi: 10.1186/s40462-022-00306-9 (PMC8864925; doi:10.1186/s40462-022-00306-9)

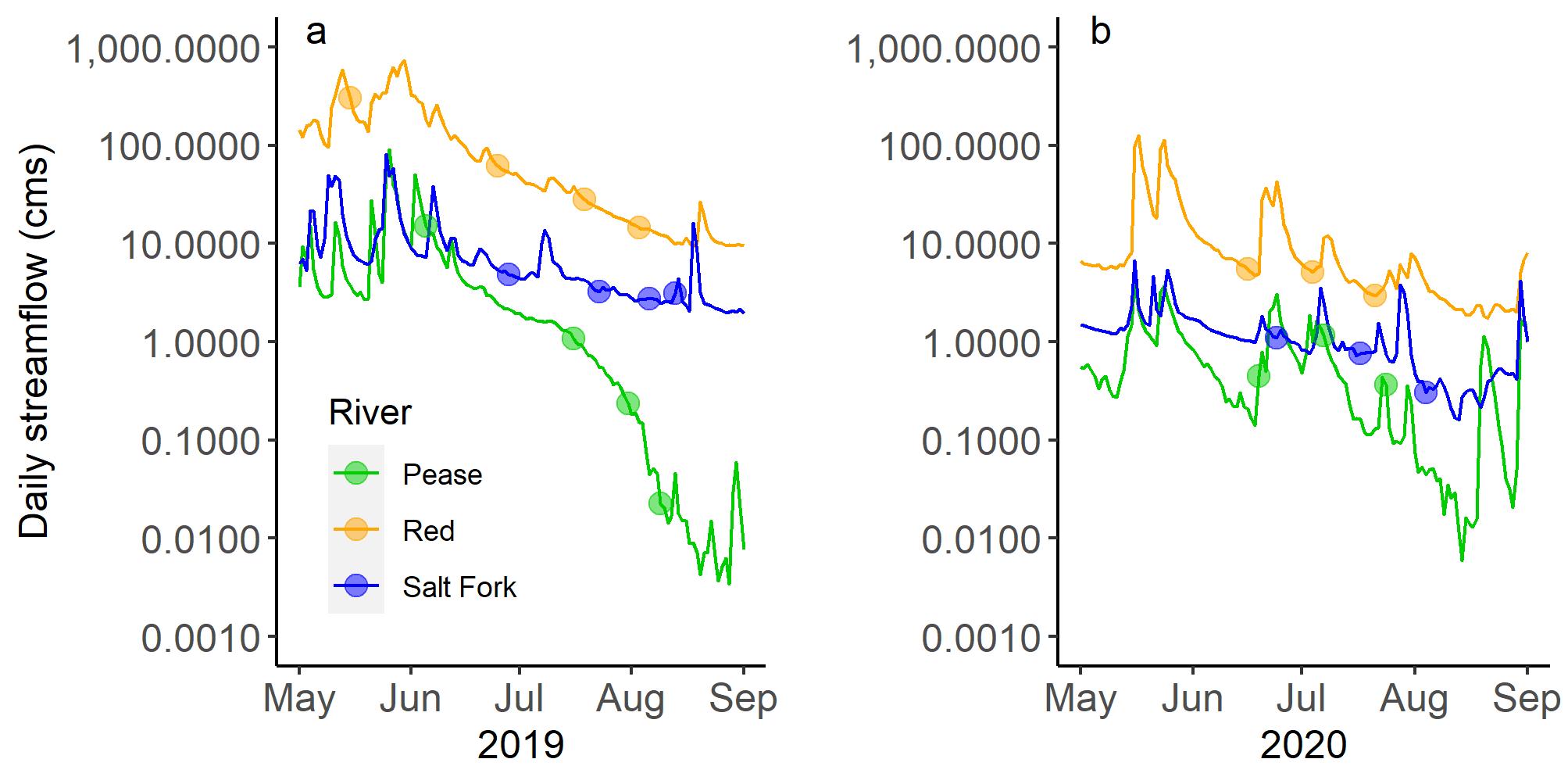

Supplement: Supplementary file 1 — Additional file 1. Hydrographs for the Red River (U.S. Geological Survey gage # 07308500), Salt Fork Red River (USGS gage # 07301110), and Pease River (USGS gage # 07308200) indicating daily discharge from May through September of (a) 2019 and (b) 2020. Lines are colored by river and similarly colored points on lines represent sampling dates. Note the y-axis is shown on a log10 scale. [file 40462_2022_306_MOESM1_ESM.jpg]

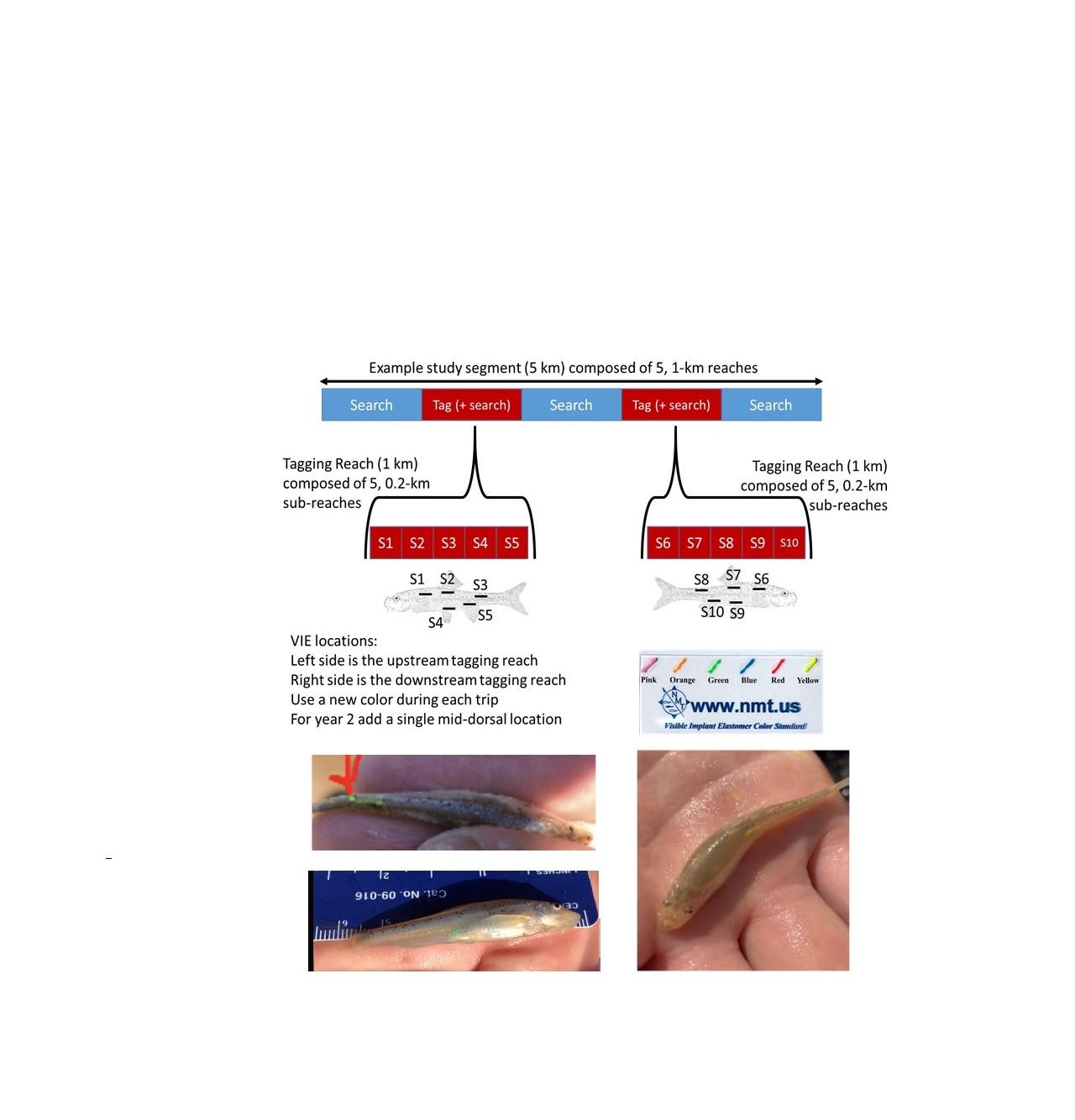

Supplement: Supplementary file 2 — Additional file 2. Conceptual diagram for a visible implant elastomer (VIE) mark-recapture study to assess movement of Prairie Chub. On each of three tributaries, we established a study segment that was 5 km in length. The study segments comprised 5, 1-km reaches. Every other reach was a search or tagging reach, in which fishes were either only searched for (i.e., search reach) or tagged and searched for (i.e., tag reach). Each tag reach comprised 5, 0.2-km sub-reaches, and within these sub-reaches fish were batch marked with sub-reach-specific body locations. This allowed greater resolution of fish movement during recaptures. During each tagging trip, a new color of VIE was used so that time at large (days) for recaptured individuals could be calculated and associated with a particular date. Picture of VIE tags are from Northwest Marine Technology Inc. and the black and white chub picture is from Everman [82]. There are examples of tagged prairie chub with various VIE colors. [file 40462_2022_306_MOESM2_ESM.jpg]

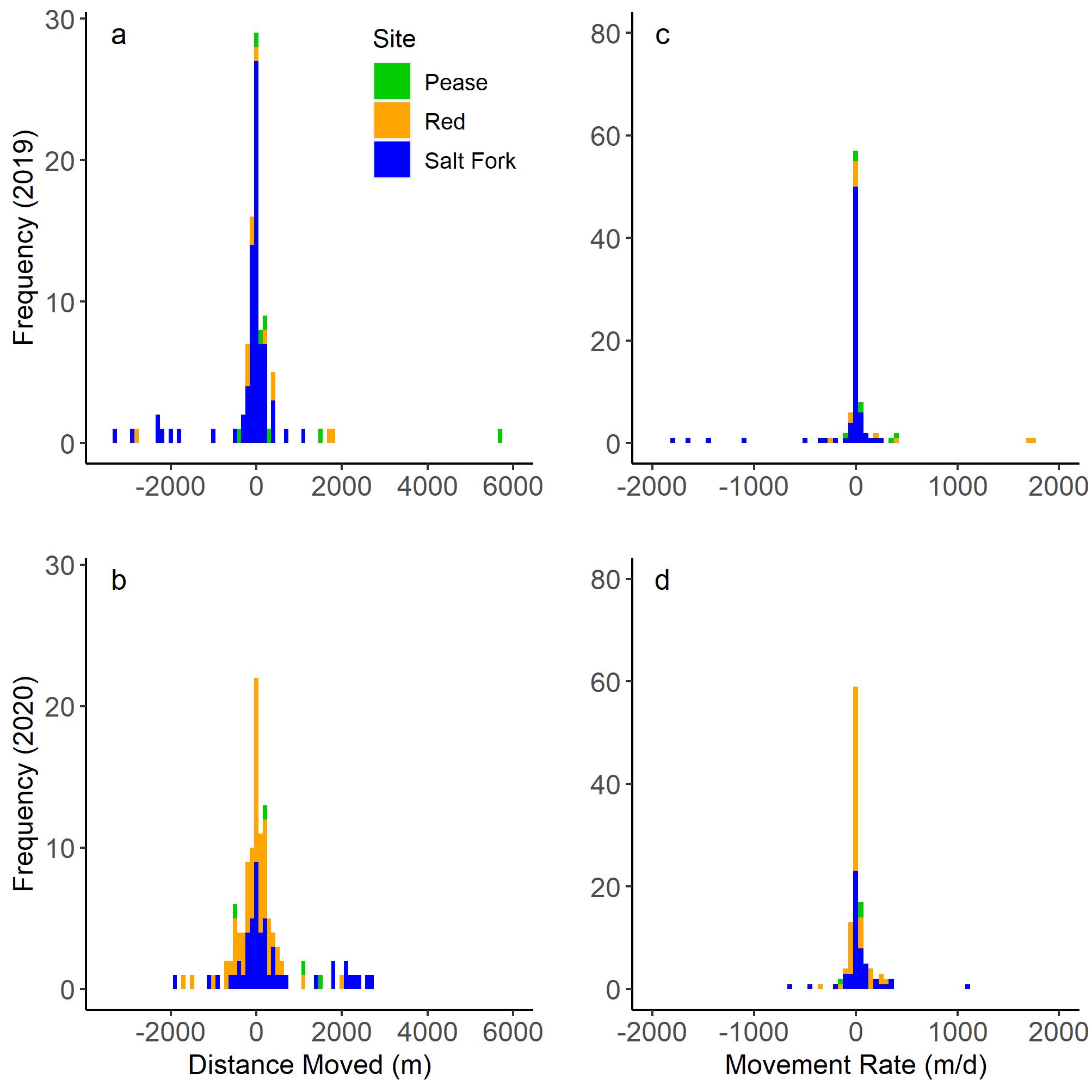

Supplement: Supplementary file 4 — Additional file 4. Frequency histograms of prairie chub movement distance (m) and rate (m/d) for 2019 (a, c; n = 94) and 2020 (b, d; n = 119). Colors correspond with fish recaptured in the Pease River (green), Red River (orange), and Salt Fork Red River (blue) and are shown as stacked bars. Negative values represent downstream movement and positive values represent upstream movement. Distance moved is shown using 100 m bins and movement rate is shown using 50 m bins. [file 40462_2022_306_MOESM4_ESM.jpg]
